# Long-term follow-up after the treatment of impacted canines in the maxilla causing severe root resorption of the lateral incisors: two case reports

**DOI:** 10.1186/s12903-024-04275-w

**Published:** 2024-04-20

**Authors:** Ning LI, Liu YANG, Qian YANG, Hongning WANG, Xiaolin XU, Tiejun WANG

**Affiliations:** 1 Department of Orthodontics, the Affiliated Yantai Stomatological Hospital, Binzhou Medical University, Shandong, Yantai, 264000 China; 2 Yantai Engineering Research Center for Digital Technology of Stomatology, Shandong, Yantai, 264000 China; 3 Characteristic Laboratories of Colleges and Universities in Shandong Province for Digital Stomatology, Shandong, Yantai, 264000 China; 4 Department of Prosthodontics, the Affiliated Yantai Stomatological Hospital, Binzhou Medical University, Shandong, Yantai, 264000 China; 5 Binzhou Medical University, Shandong, Yantai, 264000 China

**Keywords:** Impacted canines, Root resorption, Orthodontic treatment, Case report

## Abstract

**Background:**

Root resorption of adjacent teeth due to impacted canines is common, and orthodontic treatment often leads to secondary resorption or even loss of adjacent roots. Clinical reports of long-term stability after treatment are rare.

**Case presentation:**

This study reports two cases of maxillary impacted canines resulting in severe root resorption of the adjacent lateral incisors. Surgical exposure, orthodontic retraction, and alignment of the impacted canines were successful in both cases, and the resorbed lateral incisors were stable with no significant loosening and normal pulp vitality after treatment and at the 5- and 10-year follow-up appointments.

**Conclusions:**

Light orthodontic force may be used to move adjacent teeth with root resorption due to tooth obstruction. The path and direction in which the teeth are moved must be specifically designed so that the adjacent roots are not resorbed and so long-term stability can be achieved.

## Background

Impacted canines are clinically common, with an incidence of approximately 1%-3%, and the incidence of palatal interruptions is higher than that of labial interruptions [1–3]. Impacted canines affect the patient's dental function, aesthetics, and psychological health. Root resorption of adjacent teeth is more common among patients with its associated conditions. The rate of root resorption of adjacent lateral incisors is approximately 44.5%, the rate of root resorption of nonadjacent lateral incisors is approximately 7.35% [4, 5], and there are more Asians who experience severe root resorption [6]. The causes of root resorption of adjacent incisors in patients with impacted canines remain unclear and may involve factors such as sex, inflammation, location of interrupted cuspids, and mechanical pressure [7, 8].

However, diagnosing incisor root resorption is challenging due to the absence of symptoms, especially when the crown of a canine overlaps the root of a neighboring toot on panoramic radiographs, and delaying patient consultation often leads to severe root resorption, increasing the time and cost of treatment. Identifying and diagnosing impacted canines early is critical [9].

When treating resorbed lateral incisors, various factors, such as age, complaints, and severity of resorption, need to be considered. Due to the low long-term retention rate and the time and cost of treatment, it is often recommended to extract more severely resorbed incisors and restore them through cuspid reshaping or implantation [10–12]. There are few reports in the literature on long-term follow-up after orthodontic treatment of severe root resorption of lateral incisors due to impacted canines, and analysis of long-term outcomes can guide to support orthodontic therapy planning.

## Case presentation

### Patient 1

**Fig. 1:**
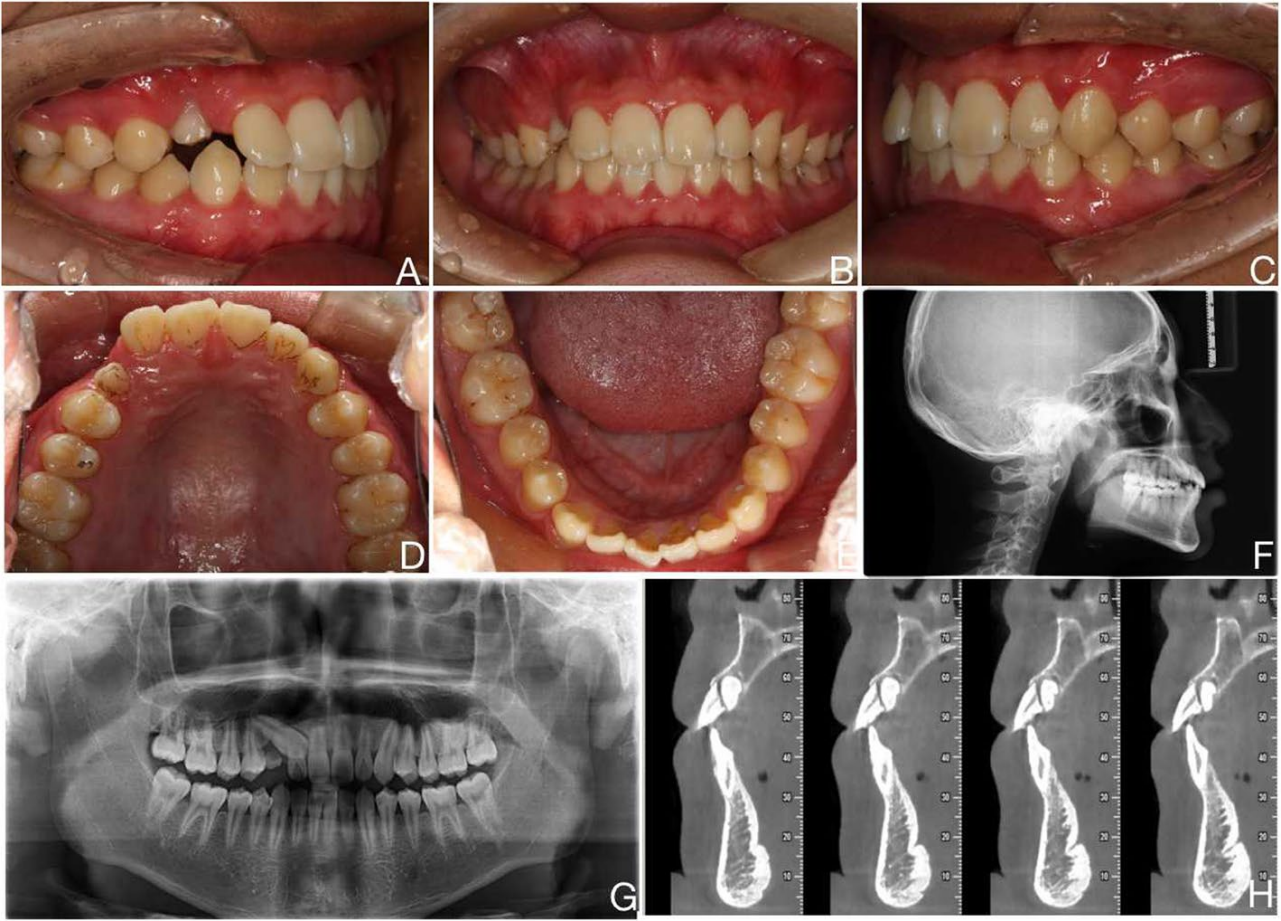
Pre-treatment oral photograph and radiograph

A 15-year-old male complained about a retained maxillary right deciduous canine. He denied having a systemic disease or family history. Facial photographs showed a deviation of the maxillary midline from the facial midline and a deviation of the maxillary midline 1.5 mm to the left. The frontal view showed mandibular asymmetry, with a 2 mm deviation of the mandibular midline to the right. Intraoral examination revealed an Angle Class III molar relationship with an overjet of 2 mm and an overbite of 3 mm. The right maxillary deciduous canine was preserved and showed grade III looseness. The right maxillary lateral incisor had a normal crown colour, grade I looseness, no sensitivity to hot or cold, and a normal pulpal electrical vitality test (Fig. 1A-E). To confirm the diagnosis, a cone-beam computed tomography (CBCT) scan was performed. All CBCT images were obtained using a CBCT device (New Tom VGI, Italy, tube voltage: 110 kV and exposure time: 3.6 s). The CBCT scan revealed a right maxillary canine with proximodistal obstruction, and crown deviation palatally, causing close contact with the lateral incisor root and resorption of the lateral incisor root to the cervical portion of the tooth (Fig. 1G and H). Cephalometric analyses indicated skeletal Class I malocclusion(A point nasion B point [ANB] angle, 1.3°) (Fig. 1F). Severe resorption of the root was observed in the right maxillary lateral incisor. We recommended two alternative options to the patient. The first option takes into account the severe root resorption of the lateral incisors, which affects the movement path of the canines and requires extraction of the lateral incisors and retained primary teeth before traction of the canines and orthodontic adjustment of the gap for adult implantation. The second option is conservative treatment-not extracting the lateral incisors and waiting for them to fall out before implantation. However, orthodontic treatment is very likely to cause them to fall out, and the patient may choose this option.

**Fig. 2:**
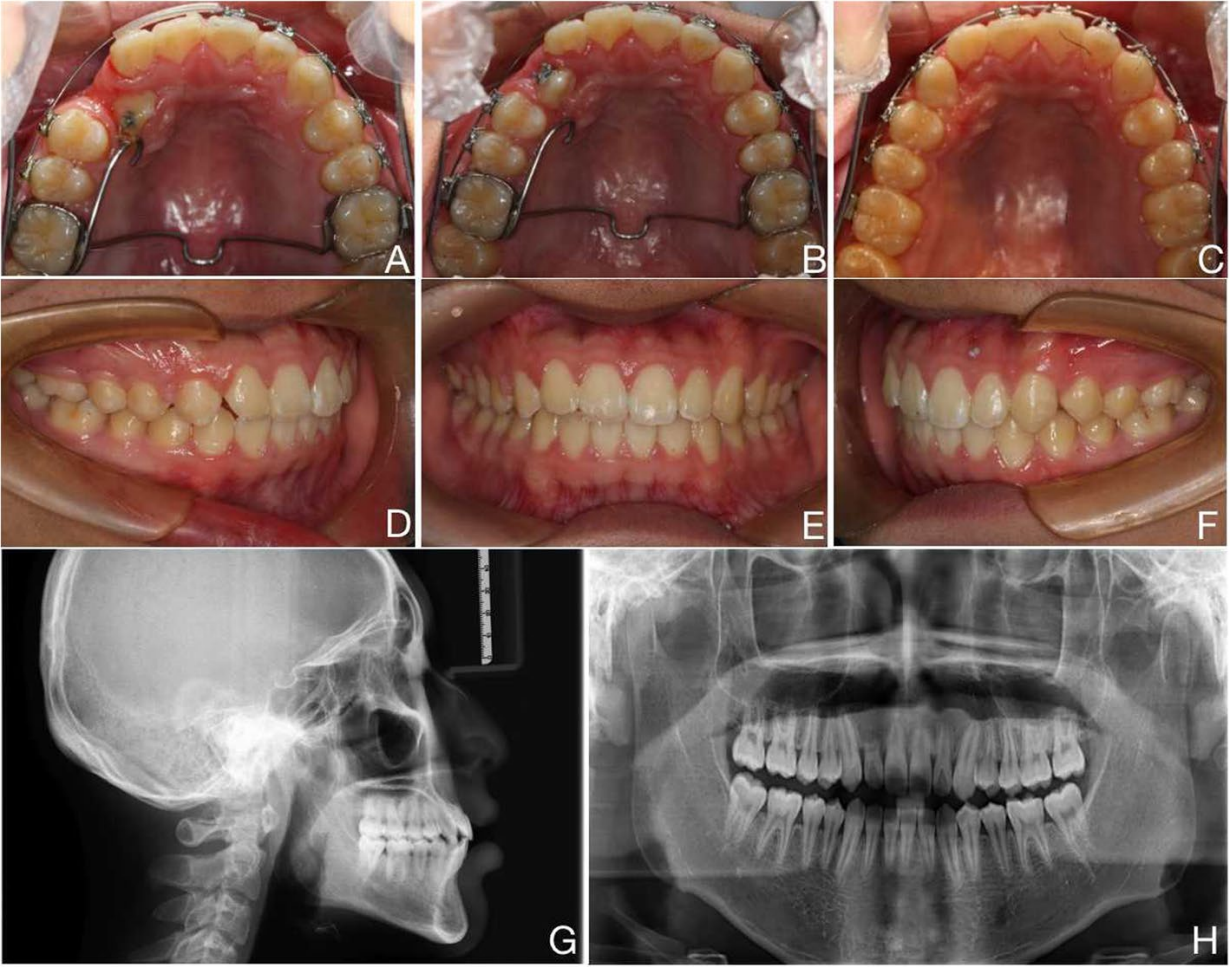
Intraoral images during treatment, intraoral images, and radiographs after treatment

Orthodontic treatment was performed with straight-wire appliances (0.022″ slot, MBT, Xinya, China) and aligners combined with a modified palatal bar retractor. As the impacted canine moves forward, the increased negative torque of the bracket helps prevent the crown from tilting too far. To avoid further root resorption of the central and lateral incisors, the adjacent lateral incisors were temporarily disengaged from the retainers during the retraction of the impacted canine, and the impacted canine was retracted and moved towards the distal palatal centre to avoid contact with the roots of the adjacent teeth (Fig. 2A and B). Orthodontic alignment was performed with serial nickel‒titanium wires. The lateral incisor brackets were cemented after the impacted canine was fully aligned into the dentition, and alignment was started with light force using fine wires(Fig. 2C).

The total duration of the orthodontic treatment was 20 months. No significant pulpal tenderness was noted in the adjacent lateral incisor during treatment, and there was grade II looseness during treatment. After treatment, the impacted canine was successfully aligned into the dentition, the pulpal electrical vitality test of the adjacent lateral incisors was normal, and the degree of looseness was grade I (Fig. 2D-F). The maxillary and mandibular midlines were in concordance, and the molars exhibited an Angle I relationship (Fig. 2G). The lateral appearance improved. Panoramic radiographs revealed that the root of the treated right maxillary incisor was essentially the same length as before treatment (Fig. 2H), and the canine root showed no significant resorption. To prevent occlusal trauma in the anterior teeth and adaptive adjustments, Hawley retainers were used for 2 years instead of clear retainers. This is because clear retainers may lead to posterior intrusion and deepening of anterior overbites. It is important to use the appropriate type of retainer to avoid any potential negative effects on the lateral incisor.

**Fig. 3:**
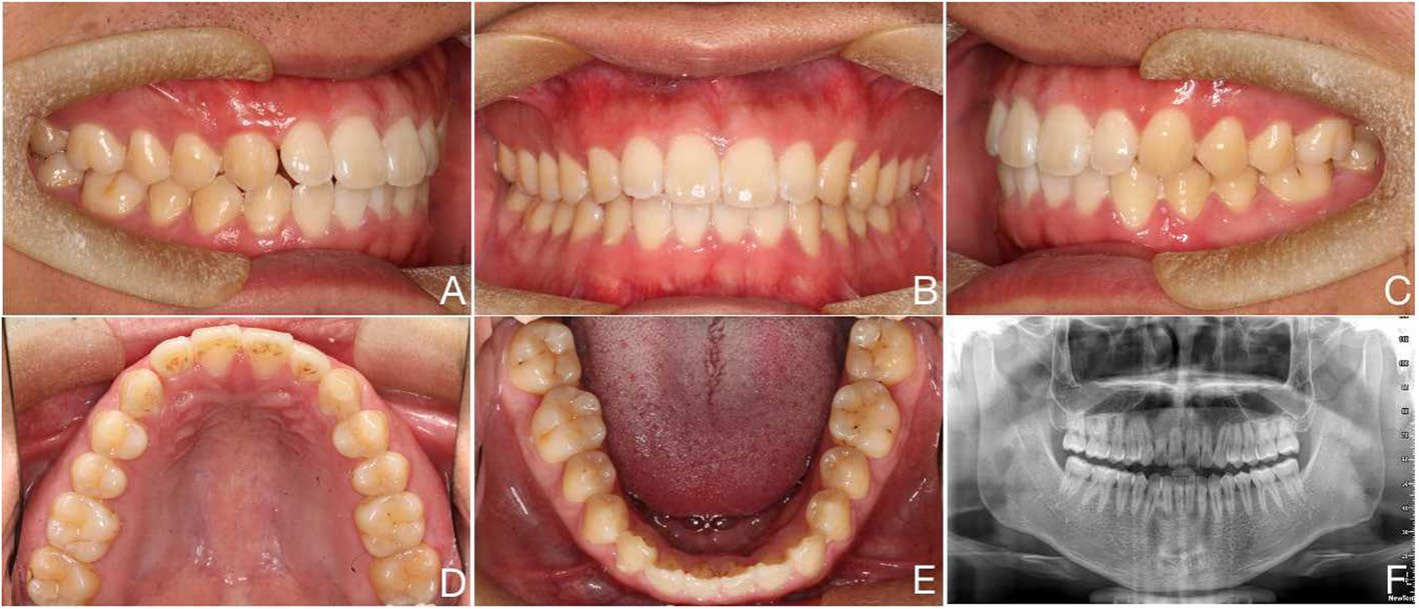
Follow-up 10 years after treatment with intraoral images and radiographs

At the 10-year follow-up, the patient's occlusion was stable (Fig. 3A-E). There was no significant loosening of the maxillary right lateral incisor and the pulpal electro-vitality test was normal. Panoramic radiographs showed that the root of the lateral incisor was essentially the same as at the end of orthodontic treatment (Fig. 3F).

### Patient 2

**Fig. 4:**
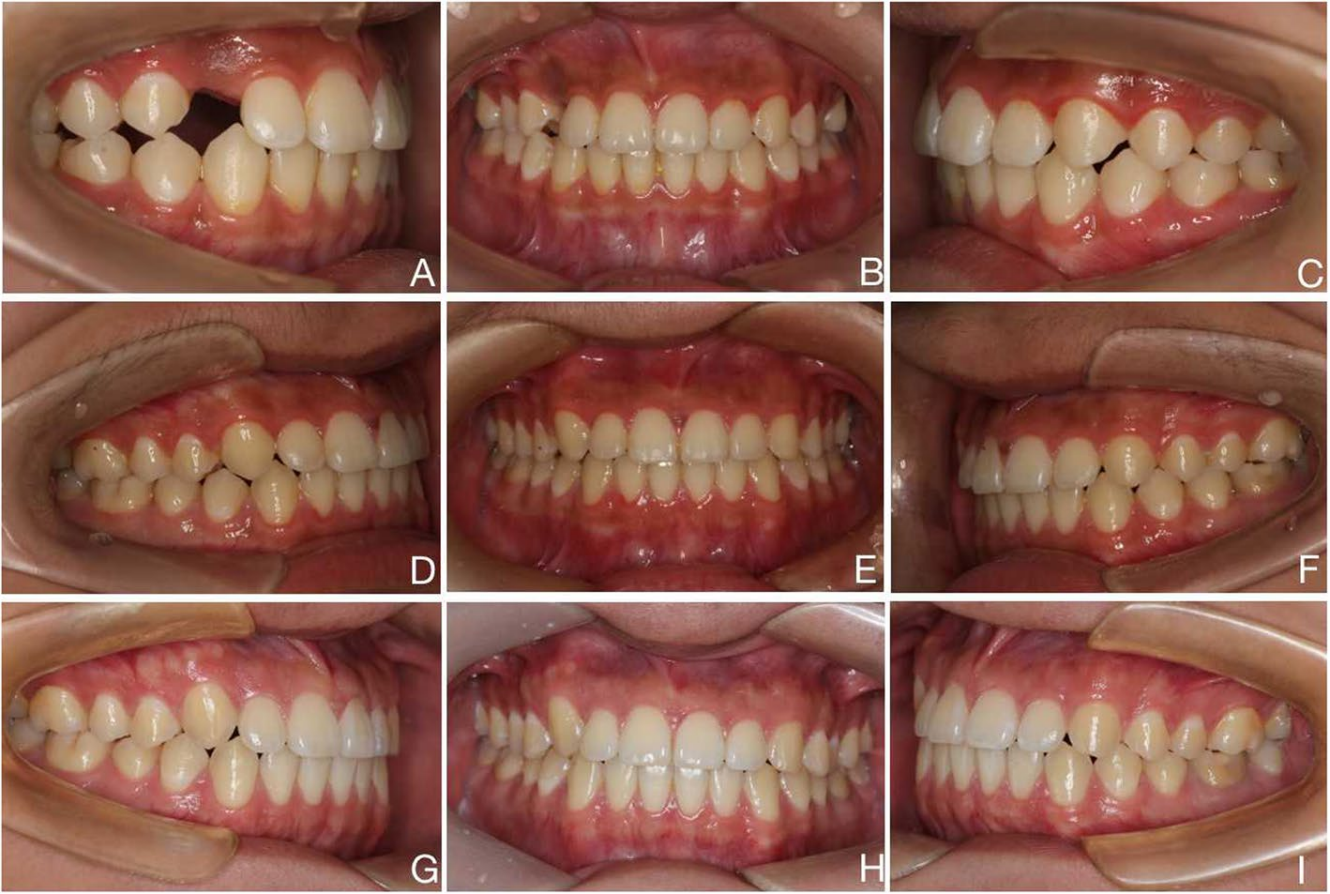
**A** to **C**. Before treatment **D** to **F**. After treatment G to I. 5-year retention

**Fig. 5:**
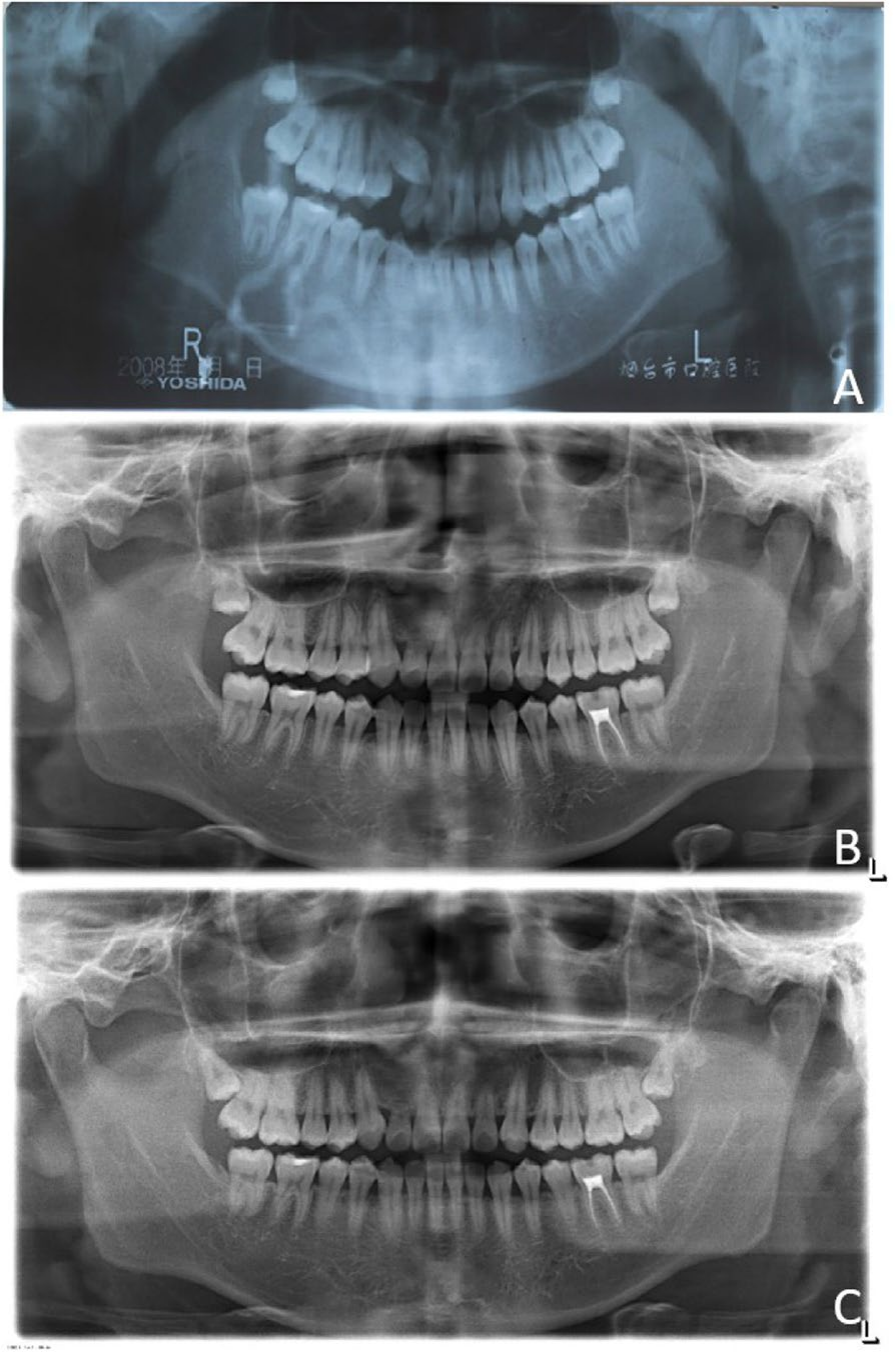
**A**.Before treatment **B**.After treatment **C**.Retention for 5 years

A 14-year-old male complained of an aesthetic defect of the upper right canine. Systemic disease and family history were ruled out. The patient had an almost symmetrical face and a straight facial profile. The maxillary midline deviated 0.5 mm to the right. A Class II molar relationship on the right side and a Class I molar relationship on the left side were observed with an overjet of 2 mm and an overbite of 1.5 mm. The right upper canine was not visible intraorally, the gap was reduced, and the right lateral incisor was labially distended, showed grade I looseness, and was not sensitive to hot or cold (Fig. 4A-C). Similarly, to confirm the diagnosis, a panoramic radiography scan was taken by a device (New Tom GINAO, Italy, tube voltage: 76 kV and exposure time: 13.6 s). The image showed a proximal mesial obstruction in the right upper canine. The crown was in close contact with the root of the lateral incisor on the labial side, and a low-density shadow of the cyst was present around the crown. Root resorption of the upper right lateral incisor exceeded 1/2 of the root length, with less periapical alveolar bone remaining (Fig. 5A). Due to the patient's fear of tooth extraction, conservative, non-extraction treatment was planned. The patient was informed of the risk of exacerbated root resorption or even loss of the adjacent lateral incisors during retraction of the impacted canine. Orthodontic treatment was performed with straight-wire appliances (0.022″ slot, MBT, Xinya, China) combined with a modified palatal bar retraction appliance. To avoid lateral incisor root resorption, the impacted canine was moved towards the labial distal centre, and the lateral incisors were not bonded to the brackets during retraction. After the extraction of the impacted canine, the patient did not show any symptoms of pulpal stress or pain.

A total of 16 months of orthodontic treatment were completed. After treatment, the maxillary and mandibular teeth were in alignment, the pulpal electrical vitality test of the adjacent lateral incisors was normal, and there was grade I. The maxillary and mandibular midlines were aligned, and the molars showed an Angle Class I relationship (Fig. 4D-F). Panoramic radiography revealed that the roots of the treated maxillary right lateral incisor and canine were essentially the same length as before treatment (Fig. 5B). Hawley retainers are typically used for two years following orthodontic treatment.

Five years later, an orthodontic clinical examination revealed stable occlusal relationships, normal colour of the lateral incisor crowns, normal pulpal electric vitality, and no significant loosening (Fig. 4G-I). Panoramic radiography showed that the long-term results after orthodontic treatment were stable, with the height of the alveolar bone around the lateral incisors and the root length essentially the same as those at the end of treatment (Fig. 5C).

## Discussion

The diagnosis of adjacent lateral incisor root resorption due to canine obstruction is often delayed in the early stages, and even when resorption reaches the pulp, symptoms are often not obvious. The rate of accurate diagnosis of adjacent root resorption on radiographs is only 30% to 50% and up to 70% on CBCT [13–16]. Misdiagnosis on radiographs is often one of the medical causes of root resorption of adjacent teeth in patients with impacted canines. CBCT provides the specific location of the impacted canines and provides the precise location for surgical exposure and orthodontic traction direction [17], thereby preventing medical root resorption caused by orthodontic therapy.

In patients with severe root resorption of the lateral incisors due to impacted canines, extraction of the lateral incisors followed by replacement of the canine and subsequent restorative reshaping is often chosen for long-term preservation [12, 18]. However, canine restorative reshaping requires removing a large amount of normal dental tissue, even requiring root canal treatment, and is not permanent, thereby increasing the burden on the patient. The two patients described in this article were young; if their lateral incisors were extracted, they would need implants in adulthood, but the lack of bone in the anterior region may lead to implant failure, as well as aesthetic and psychological consequences. There is a risk of treatment failure for impacted canines. Therefore, a non-extraction-retained lateral incisor design was used to maintain patient aesthetics and to preserve the missing tooth space for restoration in adulthood, as both patients were straight-faced, and the orthodontic design did not require extraction to orthodontically retract the anterior teeth.

With an increased risk of root resorption of the lateral incisors, impacted canines in permanent dentition often require surgical exposure combined with orthodontic treatment [19]. Rational biomechanical and directional design of traction of the impacted canines is essential to prevent root resorption of the lateral incisors [20]. The earlier an impacted canine is detected, the simpler the treatment modality. The treatment principles for both cases included a rational anchorage design, three-dimensional directional control, sufficient eruption space for the canines, and the application of light force to the lateral incisors. In these two cases, molars were used as anchorage and to prevent further root resorption caused by neighboring teeth. The impacted canines were initially designed to move palatally and distally medially, followed by labial movement after the canine crowns moved away from the roots of the lateral incisors, similar to the direction of movement reported by Herav et al. [21]. The lateral incisor brackets were then bonded after the canines were aligned to avoid any unnecessary movement of the lateral incisors. Light force was applied to align the lateral incisors. With a proper mechanical design, medical secondary resorption of the roots of the adjacent teeth of the mobile teeth can be effectively avoided.

Long-term follow-up reports of the retention of severely resorbed roots are rare. In this paper, in two cases, severely root-resorbed lateral incisors with normal orthodontic tooth movement were shown to have good long-term stability after treatment. It is possible that orthodontic treatment changed the long-term pressure of the impacted canines on the adjacent teeth, modified the periodontal tissues around the resorbed roots, and ensured the long-term stability of the occlusion. Becker et al. [22] conducted a long-term follow-up study, and orthodontic movement did not aggravate the loosening or discoloration of adjacent teeth with severe root resorption due to impacted canines, and splinting was not required after the treatment. Moreover, orthodontic movement did not increase the risk of secondary root resorption. Furthermore, Falahat et al. [23] reported that severely resorbed incisors were stable long-term, with normal long-term pulp vitality, and required no root canal treatment. The long-term observations reported in two cases suggest that a conservative, non-extraction treatment option is available for patients with severe root resorption due to impacted canines.

In this study, two patients with severe lateral incisor root resorption due to impacted canines were followed over a long period and showed good stability and normal pulp vitality 5–10 years after orthodontic treatment. For patients with high aesthetic demands for their anterior teeth, neither prosthetics nor nonextraction treatment is recommended for adolescents. Patients with severe root resorption of adjacent teeth due to impacted canines can try to retain them, but orthodontic treatment with light and intermittent force should be applied. Long-term follow-up after treatment is required to ensure success.

## Data Availability

Not applicable.

## Abbreviation

CBCT: Cone-beam computed tomographic
